# Enduring and the horizon of repair: French Caribbean post‐stroke rehabilitation amid health inequity

**DOI:** 10.1111/maq.70042

**Published:** 2025-12-17

**Authors:** Raphaëlle Melissa Rabanes

**Affiliations:** ^1^ Department of Anthropology University of Illinois Urbana‐Champaign Urbana USA

**Keywords:** Caribbean health, disability, horizon of life, racial health disparities, stroke rehabilitation

## Abstract

Drawing on ethnographic research with patients and therapists in post‐stroke rehabilitation, this article explores how Guadeloupeans strive to exist on their own terms amid postcolonial health inequities, forms of marginalization and institutional disrepair. I argue that French territorial health inequities must be understood in relation to colonial health inequities and reveal the long history of socioracial stratification in the French Caribbean. I then turn to the experience of a patient to examine how she confronts the limitations of her life chances. As she and other Guadeloupean stroke survivors push back against the contours of life delineated by systemic issues, they exist in close engagement with the horizon of life, in a movement I propose to call enduring.

## INTRODUCTION

An afternoon like many others in the readaptation clinic of the University Hospital of Guadeloupe, I shadowed Julie, a speech therapist and Afro‐descendant Guadeloupean woman in her early forties. I observed her session with Mr. D.,^1^ an Afro‐descendant man in his fifties who developed aphasia after a stroke. His aphasia meant that he lost the ability to conjure words that he previously knew, for things he still recognized. During the session, Julie and Mr. D. worked together to improve his word recollection. In one instance, she pulled the image of a strawberry out of a picture book and asked him to name the fruit. Mr. D struggled and hesitated for a moment, then looked up mischievously: “This, if I went into a market and asked a seller for this, she would tell me: you're crazy! We don't have this here! You can only find this in France, or maybe at the supermarket!” Out of the tens of images the speech therapist sifted through during the session, many represented plants, animals, or things that did not exist in Guadeloupe—a daffodil, a pine tree, an igloo.

After the session, Julie told me that she tried to select the images and words of as many local objects as possible. Guadeloupean patients who experienced aphasia after a stroke or traumatic brain injury recalled words of objects from their daily lives long before others, and they often remembered them in Creole rather than French. Yet the manual she used in this session and most of her work material came from France. Even though she grew up on the archipelago, she had to leave Guadeloupe to pursue training in hexagonal France. 2 Julie and other speech therapists tried developing an assessment scale in Guadeloupean Creole. Their efforts faltered when they obtained no assistance from the Regional Health Agency^3^ to scale the test they created.

***

To briefly situate Guadeloupe: This Caribbean archipelago was developed as a French plantation society through enslavement in the 17th century. Today, the large majority of its 370,000 inhabitants are of West African origins, descendants of those enslaved and brought through the middle passage. After the second and final abolition of slavery in 1848, freed people claimed French citizenship but remained colonial subjects for a century. In 1946, rather than decolonization by way of independence, Guadeloupe followed the course of full political integration with the hopes that this would improve access to French infrastructure and lead to equality. Today, it remains non‐sovereign, despite an important Guadeloupean nationalist movement since the 1970s (Bonilla, 2015; Camal, 2019). In addition to the majority Afro‐descendant population, “zendyens” (descendants of indentured workers brought from India to work on the plantations after the abolition), as well as “blancs créoles” (white European descendants of early colonizers), are understood to be Guadeloupeans. By opposition, those not born on the archipelago are designated as “métros” or “métropolitains” (predominantly white people born in France or Europe) or by their country of origin (with notable xenophobic undertones) when they stem from other Caribbean spaces. These categories and many more remain and are used on a daily basis to interpret social dynamics and ascribe people to distinct worlds (Gordien, 2015; Sainton, 2009).

My relationships with interlocutors are inevitably laden with these categories. I am the multiracial child of a white Frenchman and a multiracial mother, first of her family born in France, with roots in Albania, Ethiopia, and Greece, as well as Guadeloupe by adoption. My Guadeloupean great‐grandfather played an important role in my upbringing and led me to want to do research in Guadeloupe. I am read overall as *métro* yet also questioned about my origins. When I share my family history, Guadeloupeans often state that I am “Guadeloupean by adoption” or highlight that they “can see that I am not fully French,” a fact complicated by my move to the United States for my PhD.

***

Over nearly a year and a half, I followed Julie, Mr. D. and many patients, therapists, doctors, nurses, and other health workers in the neurological readaptation clinic of the sole University Hospital Center of Guadeloupe. The clinic was opened by a Guadeloupean doctor who wanted to bring post‐stroke treatment to the Guadeloupean population. When I approached the clinic about doing research on how relationships of care at the hospital were impacted by the presence of history and postcolonial dynamics, I wondered if people in managerial positions may prefer to skirt the topic. Instead, they readily engaged my research question and welcomed me as a full‐time intern. The readaptation clinic was then the main site of access to neurological readaptation^4^ for Afro‐descendant Guadeloupeans of all socioeconomic backgrounds, as well as for people from nearby islands. In contrast, white patients more likely to have supplemental insurance tended to skirt this clinic.^5^ I contend that these dynamics are embedded within a larger health infrastructure that still bears the burden of colonialism and slavery's afterlives.

I opened this article with the difficulties encountered by Julie and Mr. D. to show the weight of postcolonial dynamics in Guadeloupean neurological readaptation. When patients and therapists wrestle with the rift between how readaptation has been designed in France and the necessary accommodations to local life, they also wrestle with imperial legacies. Therapists trained in the French biomedical system have limited tools to adjust readaptation to the realities of Guadeloupean life, even when they desire to do so, and even if they are themselves Guadeloupeans. Patients in Guadeloupe repeatedly confront a system regimented by administrative, biomedical, diagnostic, and linguistic logics inherited from French national instances more than 4000 miles away. They also face health inequities, forms of marginalization and institutional disrepair that can be understood jointly as postcolonial legacies and systemic racism, even in the absence of interpersonal racism. As I will examine with the experience of Gabrielle, whom I met while she was undergoing readaptation after a stroke, Guadeloupean patients constantly strive to exist on their own terms while struggling to escape the contours of life delineated by systemic issues. I name *enduring* this constant movement of struggling and persisting, despite a sense of foreclosed future.

My theorization of enduring is built in conversation with the vocable of endurance developed by anthropologists, Black feminists, critical theorists, and postcolonial scholars. Endurance is a necessity for many people under conditions of structural inequity. Faced with medical disregard, endometriosis sufferers develop “narratives of endurance” before diagnosis (Markovic et al., 2008). In the midst of Australia's late liberal violence, Indigenous people “must find a way of enduring—of capacitating the will to endure” while “striving to change the condition in which this perseverance occurs” (Povinelli, 2011, 112, 103). In a different colonial setting, Ruiz attunes to the “enduring body” in Puerto Rican performance art, to theorize “the always enduring Rican subject” as a political mobilization of Heidegger's “enduring through time,” here a pursuit of liberation in the face of coloniality (Ruiz, 2019, 2, 9, 18). As I'll develop later when I examine the foreclosure of horizon with Ruiz, the friction between structural conditions and longing beyond them is at the heart of endurance. Yet it is essential to refuse the idealization of endurance. Ethnography, Wool and Livingston argue, is particularly apt at attuning to states “in which life goes on and on amid the damage,” while at the same time critically examining the “political romance of human endurance and resilience” (Wool & Livingston, 2017, 7, 8). Wool emphasizes the “stuckness” of this “undesirable present” where people make due amid protracted “uncertainty that seems so relentless it becomes ordinary” (Wool, 2017, 80). Dwaipayan Banerjee, also confronting the “durable consequences of long‐standing precarity,” turns to endurance in India as “an attunement, attachment, and attention to the present” (Banerjee, 2020, 172, 175). Finally, and perhaps closest to my own theorization, Jennifer Nash reflects on endurance as she theorizes “slow loss,” a Black feminist experience marked by “one's slippery and always‐contingent sense of self constituted by being in the midst of something of unknown duration and of unknown outcome,” where the political and intimate are intertwined (Nash, 2022, 9).

In this genealogy of scholars grappling with the “stuckness” of the postcolonial present, I see enduring as a way of living with present conditions despite a foreclosed horizon. I think of enduring beyond the striving for a future political transformation, an imagined moral quality of endurance or the ability to endure. Rather, I propose to think of *enduring* as a persistence that is at the same time a recognition of limitations, of structural disregard, and an affirmation of the value of subaltern existence. Contrary to an approach of endurance as striving, will, or hope in a transformed future, I argue that people constrained to the movement of enduring persist in being, even if they can no longer envision transformation.

In this article, I first investigate the structure of postcolonial health in Guadeloupe. I then turn to historical studies about racial health inequities to reveal the long history of socio‐racial stratification in the French empire. Finally, I turn to the experience of a patient as she confronts the limitations of her life chances and reckons with the horizon of her life.

Thinking through the movement of enduring, I ask: What do people desire or aspire to when the present seems daunting? How do people affirm alternate modes of life, even when they can't be fully realized? What strategies do Guadeloupeans deploy to reclaim life when their horizon is framed by structural inequities?

## RISK FACTORS: FROM READILY AVAILABLE EXPLANATIONS TO ROOT CAUSES

In the early months of my fieldwork, I was invited to observe a therapeutic education session on “risk factors for strokes” by Hélène, a white occupational therapist who came from hexagonal France and had worked at the readaptation clinic for many years. In preparation for the session, I introduced myself and my research individually to each of the patients in the hallway before asking them whether they consented to my observation. I explained that I was a medical anthropologist studying the presence of history and how it affects the experience of readaptation and the relationships between patients and health workers in Guadeloupe.

One of the patients, Mr. M., an Afro‐descendent Guadeloupean, seemed particularly interested in my research. After we all settled in the room where the session would take place, he asked me: “Do you know why Guadeloupeans have more strokes than *métros*, here in Guadeloupe and in the metropole? Is this genetic, is this the way we eat, or is this something else?”

Hélène redirected the question to them: “Do you know what factors cause strokes?”

Mr. M. responded: “There's too much of everything, so the head explodes. I lived with my mother, had problems with the neighbors, and then that day I ate salt‐fish, too salty.”

Hélène interjected: “It doesn't happen in one day. It's not because you eat too salty once. Arteries get blocked little by little…”

Mr. M. continued: “I also had stopped my cholesterol medication, so it had to happen one day or another.”

Hélène explained that some things, such as diabetes, hypertension, cholesterol, tobacco, obesity, or age, had direct effect on the arteries and others, such as stress, lack of exercise, anger or salt consumption, had an indirect effect: they contributed to increasing tension and thus had an effect on arteries, but they couldn't directly cause a stroke. The rest of the session focused on prevention and possible actions that patients could take in order to lessen their chances of having a second stroke.

Strokes are sudden stoppages of the blood flow to part of the brain. They can be of two types: The most common, Acute Ischemic Stroke, is caused by a blood clot arriving at or forming in the brain, leading part of it to be deprived of blood and thus, oxygen. Hemorrhagic Strokes, on the contrary, happen when a blood vessel in the brain ruptures, leading to a flooding that increases pressure on the brain and in the skull. Both wreak damage to the neural networks. Stroke survivors may face long‐term physical consequences, such as partial lateral paralysis or loss of function in some muscles. They also often experience cognitive changes in emotions and reasoning, sometimes memory loss or aphasia. After initial treatment in intensive care, stroke patients move to readaptation clinics where they usually undertake speech therapy, physical therapy, occupational therapy, and psychotherapy, all under medical supervision.

Strokes in Guadeloupe happen much earlier and with a higher prevalence than in hexagonal France. In 2007–2009, cardiovascular affections were the leading cause of death in Guadeloupe, while the third cause of death in France (ORSAG, 2012, 2). Within cardio‐vascular affections, cerebrovascular accidents are the leading cause of death in the region (8.7% of deaths according to Girdary et al., 2017, 177). People in Guadeloupe have strokes about 10 years earlier than in continental France. Twice as often, they die of it prematurely (ORSAG, 2018, 6). Guadeloupe is the third French region with the highest stroke mortality rate after French Guiana and Reunion (ORSAG, 2018, 1, 9). Compared to mainland France, strokes in Guadeloupe caused a 74% increased mortality rate in men, and a 45% increase in mortality rate in women in 2018 (ORSAG, 2018, 5).

These population‐level manifestations have deep roots and cannot be reduced to lifestyle choices, as often framed in public health intervention, or as natural manifestations of an imagined biological racial essence. “The slow and premature death produced from the dangerous confluence of misdirected blame and misunderstood causality is especially insidious because of how it is made to be too vast to be treatable.” (Gálvez et al., 2020, 640). Rather, medical anthropologists argue that the racialized treatment of populations—historically and to this day—leads to social and biological downstream effects. As Gravlee states, “race becomes biology” (Gravlee, 2009). Thus, in order to effect change, Leith Mullings argues that “attention to the historical and contemporary processes by which populations are sorted into hierarchical groups with different degrees of access to the resources of society shifts our analysis to racism rather than race” (Mullings, 2005, 80). This reframing leads us to examine the structuration of racial health disparities beyond the immediate question of access to health care or individual behaviors. Rather, Roberts argues, racial inequity causes health disparities, as “it makes people of color sicker in the first place—before they get to a doctor's office or a hospital emergency room” (Roberts, 2012, 333).

Hélène's review of biomedical individual risk factors didn't address Mr. M's interrogation. Had I been a facilitator of the session rather than an observer, I would not have been able to answer it either within the individual biomedical model. The root causes that lead to the higher prevalence of cerebrovascular incidents—socio‐racial stratification—remains arduous to address. Of course, having a better grasp at why Guadeloupeans have a higher stroke prevalence would be far from enough to alter that fact, as critical studies of public health and global health models of intervention have demonstrated (Adams, 2016; Biehl & Petryna, 2013). Yet, the question remains essential.

## “IN ANY CASE, WE ARE IN A COLONY HERE…” STRATIFIED CITIZENSHIP AND HEALTH DISPARITIES

A doctor engaged in stroke early intervention explained the higher mortality from strokes in Guadeloupe as an “anomaly that needs redress.” At the time of our conversation in 2016, stroke statistics were slowly improving thanks to the implementation of a coordinated pathway for neurovascular emergencies. But a combination of factors still led to increased and premature death and long‐term disability from strokes: In addition to the higher prevalence of individual risk factors such as untreated hypertension and diabetes in the Guadeloupean population, patients who suffered a stroke continued to face delays in pre‐hospital and in‐hospital pathways when any delay in treatment leads stroke sufferers to face a significant loss in opportunity for recovery. Furthermore, once in neurological readaptation, despite the dedication of all categories of health workers to work with patients as often as they could, due to a series of structural shortages in facilities, materials, and personnel, patients in recovery couldn't receive the nationally recommended level of treatment.^6^

Over 70 years after the full integration of overseas colonies into France, equality with hexagonal France still hasn't been achieved. In 2014, a French National Audit Office report on *Health in the Overseas* highlighted two paradoxes: First, “despite their geographic, human, and organizational specificities,” overseas departments and territories all have in common “persistent difficulties in health conditions.” Second, while health conditions in the overseas departments and territories are more favorable than in neighboring countries, they are also highly unequal compared to hexagonal France (Cour des Comptes, 2014, 10, 12, 48). The audit diagnosed a series of problems. It observed that France didn't fund health care in proportion to the needs of the population and that while chronic conditions were more prevalent in overseas departments, the state didn't provide adequate support for preventative public health campaigns. It also explained that overseas health care systems did not “sufficiently” respond to the effects of socioeconomic, climatic, and environmental determinants of health. The report depicted a combination of problems in outpatient care: health professional shortages and medical deserts, insufficient coordination between different actors, and high salary costs. Additionally, it highlighted issues in hospital care, such as emergency care overload and deficient management of human resources.

Following this report, the minister of health and the minister for the overseas commended a “national strategy for health in the Overseas,” with strategic plans adapted for the specific concerns of each department. This program, as well as the 2017 “Law for Real Equality in the Overseas,” have been launched to reduce the disparities in life chances between Hexagonal France and its now integrated former colonies. In the Health Regional Project for Guadeloupe for 2018–2028, the Regional Health Agency of Guadeloupe recognizes four challenges, including medical deserts and inequality in accessing healthcare, but also broader disparities in health and premature mortality linked to social determinants of health (ARS, 2018).

As these plans were being hatched, Mr. E, another patient to whom I presented my project, offered a pithy commentary on how history came to bear on his experience: “In any case, we are in a colony here…” My research questions didn't faze him. In fact, his perspective—while not shared by all—is fairly common in Guadeloupe. He went on with his readaptation program and didn't extend his observation further. This statement, made in passing, pointed to coloniality as a condition of present life. It revealed a deep history that lives just below the surface in the minds of many Guadeloupeans. It led me to turn to the archeology and history of health and medicine in Guadeloupe to grasp the long history of unequal treatment of socio‐racial groups that manifested in health disparities.

Studies of Guadeloupean colonial cemeteries have exposed the “particularly dire life conditions” of enslaved people (Courtaud & Romon, 2004, 66), revealed through bone lesions from advanced tuberculosis, malnutrition, repetitive stresses, and mistreatment (Courtaud, 2013, 13; Dutour et al., 2005). All the while, slave owners wrongly explained infectious disease outbreaks decimating enslaved populations as caused by poisoning practices rather than unsanitary conditions in slave quarters (Bougerol, 1985). Even after the abolition of slavery, the public health of Afro‐Descendant Guadeloupeans was long overlooked. An imbrication of medical training, medical knowledge, and racialized health policies harmed Afro‐descendant Guadeloupeans during the 19th century (Taffin, 1985), revealing the structural construction of racialized understandings of life value and its translation in differentiated life expectancy between racialized groups. For example, the French government covered up a cholera outbreak in 1865–66 to conceal “the miserable living and demographic conditions of the black majority” (Taffin, 1992, cited by Jennings, 2006, 73). Meanwhile, thermal spas were developed for colonizers in Guadeloupe and other French colonies (Jennings, 2006). Up until the early 20th century, Antillean medicine remained focused on an “epidemic regime,” oriented toward emergency intervention rather than prevention and was inaccessible to the majority of Antilleans (Dumont, 2011, 845). The health of Afro‐descendant Guadeloupeans only seemed to become a concern after Antilleans fought for their conscription in World War 1, and while the government of the Third Republic was trying to fortify what it called its “human capital” in the interwar period (Spivak, 1987). Public health development emerged slowly, leading to the opening of the first general hospital of Guadeloupe in 1936 (Dumont, 2009). Finally, the first large hospital structure emerged in Guadeloupe in the late 1970s and gave rise to the University Hospital Center in the 1980s.

These historical studies indicate a difference between the purported equal rights of all citizens and the inequities faced by freed people and their descendants. Access to medical care and health outcomes remained stratified by socio‐racial lines long after the abolition of slavery and even the formal end of colonial status in 1948. The 2014 audit on *Health in the Overseas* showed the persistence of health disparities and broader social disparities between overseas territories and hexagonal France into the 21st century. While my work is not an epidemiological or biocultural study of Guadeloupean health disparities and I can't trace a continuous line between different historical eras, I argue that it is important to hold this history in mind to understand the statement made by Mr. E and, more broadly, the weight of history in present‐day Guadeloupe.

In the United States, historian Saidiya Hartman calls “afterlives of slavery” the differential life trajectory of Afro descendant Americans, marked by “skewed life chances, limited access to health and education, premature death, incarceration, and impoverishment” (Hartman, 2007, 6). Racialized populations show disparities in health compared to groups who benefit from health‐promoting environments precisely because health is an indicator of people's broader social and historical living conditions. If the afterlife of slavery present in racist medical practices is more violently visible in the United States, it lives on in people's bodies, as well as health infrastructures and research practices throughout the Black Atlantic (Carter, 2021; Davis, 2019; Valdez, 2022).

While different, racialized health disparities in the United States and France have resonances. If, according to Geronimus's weathering hypothesis, African Americans experienced “earlier deterioration of health (…) comparable to that for Whites who were 10 years older” (Geronimus et al., 2006, 831, 832), in Guadeloupe, people have strokes about 10 years earlier than in hexagonal France. With Christina Sharpe and her reading by Ruha Benjamin, Tracie Canada and Chelsey Carter, I think of weathering as a manifestation of the “total climate” of antiblackness shaped by the atmospherics of slavery (Benjamin, 2022; Canada & Carter, 2024; Sharpe, 2016, 105). I now turn to the experience of one stroke patient to understand how functional and structural limitations became entangled for her, and how these came to bear on her life.

## “I WAS RIGHT TO BE WARY…” NAVIGATING CONSTRAINTS

About a year after a stroke left one side of her body paralyzed, Gabrielle—a middle‐class Afro‐descendant Guadeloupean woman in her sixties—participated in a Stroke Therapeutic Education Workshop. When patients were invited to talk about what happened to them, she described her hemiplegia as a body split in two, unrecognizable: “My right side was an empty box, and on the left it was normal, it was full.” Holding her body between emptiness, alienation, and belonging. Gabrielle quickly shifted to the present tense: “It's a handicapped [person] on the right side, and on the left side it's me.”

At the end of the workshop on the second floor of the clinic, the elevator that brought everyone upstairs no longer worked. Unsure about when it would work again, the therapists resorted to carrying down participants who couldn't walk. Four health workers set out around Gabrielle. She let herself be lifted up without a word, but watched carefully. When her wheels finally touched the ground floor, Gabrielle turned to me and exclaimed while laughing: “You'll have to put this in your book!” Soon her laughter turned to tears. What did this moment mean for her? Following the scene from atop the stairs, I too felt that the scene was important. I saw it as encapsulating the dire state of the health infrastructure and the intimacy it forced upon its conscripts.

Gabrielle's ambulance was late that day. We set up outside in the bright midday sun. Dependent upon an over‐taxed system of medical transport, she often waited and I often kept her company. My presence seemed to distract her: “What else? Ask me anything you want!” she prompted me once. I had grown to know Gabrielle quite well, first as an inpatient for 5 months, then as an outpatient when she pursued speech, occupational, and physical therapy as well as psychotherapy. Over the 15 months she attended the clinic, I followed her through her care pathway and also visited her at her home, where I met her husband and several of her adult children.

On the day of the workshop, during the hour and a half she waited for her ambulance, I asked her many questions, but eventually let the silence linger. Gabrielle turned pensive: “I didn't want to go upstairs when I saw the state of the elevator, but I had to comply. I was right to be wary… Why is my life so full of stairs?”

I had a clear picture of the other stairs that prompted her anguish: In a home visit with an occupational therapist to prepare her return, we had counted at least 20 steps to the main floor of her house. The family looked into installing an elevator, but the cost was prohibitive, and the public assistance for this home adaptation wasn't sufficient. Upon returning home, she needed to be carried by two paramedics each time she wanted to leave her house. She didn't like that the stairs constrained her to medical transport, especially since it was in plain sight of her neighbors, who could also see the ambulance in front of her home. To avoid this, she restricted her outings and only left her home for medical appointments biweekly. The stairs and her forced reliance on medical transport kept her away from the world and constrained her to her home.

## “THEY GAVE ME A MONSTER”: VALUE MISALIGNMENT, OR THE DANGERS OF AUTONOMY

If Gabrielle complained about obstacles to her mobility outside her home, she didn't complain about barriers to moving independently inside it. She only lamented needing external assistance: “I like when [my husband] helps me. He is very devoted to me. He loves me deeply and stays by my side. But he's an old man too, not strong enough to carry me, so now *strangers* come into our bedroom to put me into bed.”

Nevertheless, some of her therapists insisted on prescribing her a motorized wheelchair so that she wouldn't require her husband's help to move about. After learning to use one in the clinic, she reluctantly agreed to try it at home. In her following session, Sandrine—a white psychologist from hexagonal France who had lived and practiced in Guadeloupe for several decades—inquired: “Have you received your Rolls Royce?” Gabrielle responded without enthusiasm: “It is safely at home, but *safely away from me too*.” Despite this enigmatic answer, the psychologist didn't probe further. Instead, she instructed her about how to spare the chair's battery before moving to relational concerns about her adult children and the logistics of her life at home.

It took the delicate attention of Julie—the Afro‐descendant Guadeloupean speech therapist who showed the strawberry image to another patient in the article's opening scene—for Gabrielle to be more comfortable sharing her concerns: “This machine scares me,” Gabrielle told her point‐blank. “And it doesn't bring me anything good.” Julie immediately notified Line, an Afro‐descendant Guadeloupean occupational therapist who took care of her home adjustments. Line had long understood that Gabrielle didn't want the powerchair and promptly asked for it to be picked up and removed.

It is only a month later that Gabrielle was able to express to her psychologist the deep underlying reason of her refusal: “Sometimes, I close my eyes and I see myself pushing this [the powerchair] down the stairs. So I said: it's better to get rid of this machine than to give myself bad ideas. I would have more anxiety if I still had it at home.”

A few weeks after it was removed from her house and while I visited her there, she spoke more about what the powerchair represented: “They gave me a monster. Ever since they took it back, I am more at peace. It had big eyes. It wanted to swallow me. It frightened me a lot.” I asked her: “Because it was powerful? Because you didn't control its power?” With a smile, she tapped her manual wheelchair's armrest, emphatically: “This one can't escape me. It's not efficient!”

The motorized chair offered Gabrielle the possibility to move about her house without assistance, but it was so powerful that she feared it could escape her control, drive her to a scenario that until then only lurked in the back of her mind. The power of the chair made it possible for her to imagine throwing herself down the stairs, potentially taking her own life. Contrary to what some of her therapists imagined, it didn't improve her quality of life. Instead, it increased her anxiety and brought to the surface suicidal ideation that she had not expressed before.

Interestingly, Gabrielle was first able to share her fears and reticence about the powerchair with Julie and Line, her speech and occupational therapists, who were both Guadeloupeans and Afro‐descendant. She breached the topic of her suicidal ideation with her psychologist only a month later. And she didn't speak of her refusal of the wheelchair to her other occupational therapist. The latter two were both white and from hexagonal France.

When I asked Gabrielle why things unfolded in this way, she didn't share a clear reason: “Time had come to talk about it, I don't know why. With Julie, we talk well. She has known me for a while and supports me a lot.” Concerned, she added, “I hope they're not upset with me?”

Her worry interpellated me. Did she feel uncomfortable addressing this topic with me? Or addressing it altogether? Did she fear that her therapists would hear of it and that it may affect her care? Or did she truly not consider socio‐racial dynamics playing out in who she felt she could confide in? Since she skirted the subject, I could only take note that who she opened up to and who she worried about offending aligned with racial identities that commonly mark people in Guadeloupe.

Rather than only a matter of skin color or a protection against interpersonal racism, I posit that Gabrielle quietly pushed back against a vision of readaptation that centered around autonomy and heralded the powerchair as the ultimate technical fix. Before the motorized wheelchair, thoughts about ending her life were only thoughts that she couldn't pursue, thoughts she could dwell on without immediate danger. The appearance in her home of the machine she called “the monster” rendered palpable the possibility of harming herself.

Gabrielle's *métro* therapists pushed for the powerchair as a response to an imagined need for autonomy. What they saw as dependency on her spouse that could be partially redressed by technological aids, she saw as love and intimacy. Gabrielle's reticence contradicted what they envisioned could benefit her. They were unable to hear it, at least initially. Her Guadeloupean therapists, on the contrary, quickly understood the powerchair as another iteration of the strawberry in my opening sequence: incongruous and misaligned. This is not to say that no Guadeloupean could benefit from or want to use a powerchair. But for Gabrielle, this technical fix threatened the interdependency that kept her alive. Her Guadeloupean therapists readily recognized this core value.

Did she perceive her Guadeloupean therapists as more likely to share her values? Did the insistence of her French therapists to make her adopt the powerchair confirm a sense that they didn't *get* her? Even without demonstrated bias, the therapists’ contrasted visions of Gabrielle's needs nevertheless fell along the lines of colonial power relation.

## HORIZON FORECLOSED?

Despite her inability to hear Gabrielle's distress, her psychologist was not the cause of her grief. The readaptation team was never able to nudge Gabrielle out of stillness. In a meeting about the course of Gabrielle's treatment, her physical therapist, Nora, explained: “She is convinced that she won't recover, so she doesn't. She gives up and lets herself fall down.” In physical therapy sessions I observed, she would, for example, stop attempting to stand and carry herself on the leg that remained active. “It's as if she can't envision progress, so she abandons any movement,” Nora continued. Despite months of work, the team kept feeling that Gabrielle couldn't imagine progress, and that it prevented her from recuperating more movement.

When other patients strive to recover movement after hemiplegia or find ways to reintegrate their full selves into their transformed body, Gabrielle never seemed invested in readaptation. When half her body became paralyzed, it was as if she couldn't recognize herself fully. In losing her *gestalt*, her ability to grasp herself as a totality in motion, she lost a sense of belonging in herself. Progressively, she stopped using her functional leg and arm. As she once explained to me: “When I lost hope to reuse my right arm, I decided to stop using the left hand.”

Sifting back through my notes, I stumbled upon a jotting. When Gabrielle was in the early months of readaptation, her doctor prescribed sessions of mirror therapy. She hoped that Gabrielle, by working with the image of her functional left side in the mirror, would start envisioning movement in her right side, cultivate the cognitive imagination of her having control over her arm and leg, and eventually start moving. But instead of repeating the movements demonstrated by her physical therapist, Gabrielle sat and looked at the reflection of her arm and leg without attempting to move. I noted her silence that day, how she glanced at the reflection of her arm, then down to her leg, before looking away towards the horizon for long minutes. She seemed engulfed by a sense of stillness. In my notebook, I wrote: “Horizon foreclosed?” Instead of allowing her to glance beyond current immobility and see herself moving in a near‐future, mirror therapy led her to see her body as fully paralyzed.

In Freudian terms, a lost object, introjected, can progressively cast its shadow on the ego. If “an object loss” can be “transformed into an ego loss” (Freud, 2001, 249), for Gabrielle, it's as if the loss of the body she knew and desired shook the delineation and grounding of her life, her horizon.

“But what happens when horizons disappear?” asks Petryna (2017, 260). I didn't know yet that she developed the concept of “horizoning” to describe a recalibration of our understanding as environmental thresholds shift, in order to open up space for transformation (Petryna, 2022). I didn't know either that Sandra Ruiz theorized endurance precisely in friction with the horizon, or past it: “Endurance (…) is about laboring to eventually stare past the horizon with apprehension, longing, pain, and pleasure—no feeling invalidated by another in the long pursuit of liberation and continual existence.” (Ruiz, 2019, 12).

Gabrielle pressed against the horizon and persisted in living, but instead of a reckoning—a struggle that would somehow be productive, as in Ruiz's vision—she experienced it as a form of entrapment. Up against the horizon of her life, she didn't find space for recalibration. This is why I describe her persistence through the movement of *enduring*—an ever‐expending effort caught in a form of stillness—far from the quality of endurance, an ability to see past the horizon, or to recalibrate it. I do not praise a form of heroic resilience in Gabrielle's movement of enduring. And gesturing to Mora Bailey's “Black Feminist Disability Framework,” I invite the readers to guard against their own revival of the “Strong Black Woman” trope, whereby Black women are praised for their resilience while their suffering is erased and their disability disallowed (Bailey, 2019, 21). Rather, I write against the idealization of resilience and underline that Gabrielle grappled with a sense of caughtness in a never‐ending present. By refusing the motorized chair, she demonstrated a desire to persist, even as her world shrunk to a close‐up horizon, marred with shadows.

## ENDURING AS CONSTRAINED REPAIR

The foreclosure of Gabrielle's horizon stemmed from an interplay between her refusal of bodily transformation and structural limitations experienced through the presence of stairs and inaccessible public spaces. While most disabled people throughout the world worry about access, the interplay between body and infrastructure has specific stakes in a post‐slavery society like Guadeloupe. The afterlives of slavery play out at the embodied nexus of race, space, and place, as Black geographies demonstrate (Hawthorne & Scott Lewis, 2023; McKittrick & Woods, 2007). At the level of spatial politics, lineage and racialization still play a role in who gets to claim ownership over what land. At the level of embodiment, gender, sexualization, and racialization still carry echoes of enslavement and violence against Black women, reduced to their laboring bodies, turned into territories and “marked as decipherable and knowable” (McKittrick, 2006, 45).

McKittrick argues that this history carries over in contemporary spatial practices, where “Black women's own experiential and material geographies, consequently, indicate a very complex and difficult relationship with space, place, and dispossession” (McKittrick, 2006, 45). Alongside McKittrick, I stress that this charged relationship with space doesn't lead to an inevitable perpetuation of alienation: Black women reclaim their beings and the geographies they occupy through embodied practices.

Gabrielle shows how to sustain emplacement and belonging despite a foreclosed horizon. Hopefully, I have showed alongside her that this remains possible even when embodied practices are less visible and do not lead to reclaiming public space but rather dwelling in the everyday.^7^ I developed the concept of enduring to show the importance of this desire to persist in living even in the face of duress, and even in practices that wouldn't be readily recognized as reclaiming of space.

A year and a half after I last saw Gabrielle, I checked in with her daughter. She shared: “Her mood is not always very good, her smiles too rarely spontaneous… Each day, she awaits mercy. But she's still ok. She gladly laughs with the nurses.” Gabrielle doesn't smile on her own, yet she gladly laughs with others. Her daughter lovingly observed this paradox, echoing Christina Sharpe's wake work:

“attend to, care for, comfort, and defend, those already dead, those dying, and those living lives consigned to the possibility of always‐imminent death, life lived in the presence of death; to live this imminence and immanence as and in the ‘wake’” (Sharpe, 2016, 38).

Enduring, as Gabrielle shows us, is not holding onto hope, is not a projection into a future other than awaiting death, yet no desire to rush towards it. It is life in this in‐between space, even when the horizon is foreclosed, while relishing the meaning and joy brought by connection.

“She awaits mercy,” her daughter wrote to me. Perhaps Gabrielle herself was holding a wake to her own life, “consigned to” yet keeping at bay an “always imminent death”. And in that space near but not at death, she remained sustained by her relationships, which I also understand as the wake‐full presence and attention of others, an attention where “We, Black people everywhere and anywhere we are, still produce in, into, and through the wake an insistence on existing.” (Sharpe, 2016, 11)

Years after her stroke—and despite a constant presence of finitude she desired at times—she kept on living and “gladly laughed” with others. She persisted in living while also “await[ing] mercy,” even as she experienced structural limitations and discouragement. While she was entrapped by the fixity of space around her and how she partially abandoned her own body, Gabrielle also found ways to persist in living relationally. I see Gabrielle's laughter and her daughter's attention as embodied movements of *enduring*, an insistence on nourishing the possibility of life amid social death and near‐death. Gabrielle found movement, albeit at a different scale, when she engaged with those around her.

Rather than a story about strength, resilience, or praise for such qualities in contexts of scarcity, I offer an ethnographic reflection on *enduring*, a fraught relationship to life in the midst of limitations. The experiences of Gabrielle and other stroke patients in the readaptation clinic bring to light the broad persistence necessary in Guadeloupe, when life is structurally hindered. Far from resilience, this movement of enduring is fraught with hardship and shouldn't be glorified. Yet the efforts it entails should be recognized. I call enduring this way of living “in the presence of death” (Sharpe, 2016, 38), in close intimacy with the horizon of life. Enduring takes place in a confrontation with all‐encompassing conditions of life, the weather that shapes the layered conditions of postcolonial life, from interpersonal dynamics to institutional logics, to structural frames of existence.

In this context, persisting in living is a constrained decision. Perhaps it is also a form of constrained repair? Understanding health as not just about bodies, but about collective embodied life in places marked by history, I propose to think about repair not as the erasure of harm or return to full bodily capacity or idealized agency, but instead as a grappling with one's condition in embodied ways. Enduring—as an attempt to exist within yet despite the horizon of life set by postcolonial constraints—is perhaps the first effort to push back and stretch out against the horizon of life, at the scale of one's existence.

## ETHICS STATEMENT

Ethical approval for this research was obtained from the University of California Berkeley.

## ENDNOTES

1 All interlocutors were informed of my research and gave their consent to figure in this ethnography, but only people with whom I had sustained conversations chose a replacement name. When they didn't, I give them an initial. This choice is a refusal of the ethnographic convention of renaming people without their participation, in a context where colonial administrators assigned arbitrary names upon emancipation.

2 Hexagonal France is an iconic description of France according to its six‐sided shape, to move away from a center/periphery dynamic between mainland and overseas France. Yet the centrality of the hexagon remains: All but one speech therapists I interviewed were trained in hexagonal France, and the last one was trained in Belgium. A program in speech therapy finally opened in September 2022 at the Université des Antilles in Guadeloupe. Its first cohort of 15 is set to graduate in 2027.

3
*Agences Régionales de Santé* are regional public health agencies under the tutelage of the French Health Ministry. They are responsible for piloting the national health system in each region.

4 The team made a conscious effort to move away from an ableist model centered on normative standards. While the term *reeducation* was still used to talk about the attempt to regain as much function as possible, readaptation therapists and doctors spoke about working towards *readaptation*, adaptation to change by modification of patients’ environment or gestures.

5 The first rehabilitation doctor in Guadeloupe Dr E. Galanth talked about the “Boeing syndrome” (flying to France for treatment) as due to the lack of local rehabilitation structures before the 1980s (Villoing et al., 2016, 59). Twenty years later, I was told that people with private health care supplements turned to private clinics for treatment, on the island or in Hexagonal France. In a context where class still heavily correlates with racial stratification, this resulted in white patients choosing care outside the University Hospital in greater proportions. Of the hundreds of patients I encountered during my long‐term fieldwork in the clinic, only two were white and they were both in situation of socio‐economic distress.

6 Material conditions have changed drastically since the move of the readaptation clinic to a new center in 2018, even if difficulties in recruiting therapists remain.

7 This formulation is a gesture to Veena Das’ attention to the ordinary and call to return to the everyday (Das, 2013, 2020).
